# Competency-based, multicomponent teaching reform in medical imaging technology: a quasi-experimental study

**DOI:** 10.3389/fmed.2025.1708856

**Published:** 2026-01-12

**Authors:** Chunhua Qi, Shuli Zhou

**Affiliations:** 1 Medical Institute of Technology, Baicheng Medical College, Baicheng, Jilin, China; 2 Anorectal Surgery, Baicheng Central Hospital, Baicheng, Jilin, China

**Keywords:** colleges and universities, medical imaging technology specialty, specialty group, teaching reform, competency-based education

## Abstract

**Introduction:**

The growing demand for medical technology highlights the need to improve education in medical imaging technology (MIT). Traditional teaching presents concrete challenges, such as lecture-centric instruction, limited simulation time, and weak school–hospital coordination, which have prompted curricular reform. This study evaluated a competency-based, multicomponent teaching reform that integrated staged training, digital and simulation resources, updated methods, and school–enterprise collaboration.

**Materials and methods:**

We conducted a quasi-experimental, sequential-cohort study comprising a control cohort that received routine teaching (July 2021–June 2022; *n* = 60) and a subsequent intervention cohort that received the reformed program (July 2022–July 2023; *n* = 60). The primary outcomes were theoretical knowledge (0–100 written exams) and hands-on imaging skills, assessed using an OSCE-style, station-based practical exam (0–100). The secondary outcomes included learning ability, clinical reasoning/analysis, and problem-solving, which were measured using Likert-scale instruments aggregated to a 0–100 scale, as well as participation in competitions and research, publication rates, professional examination pass rates, employment, and satisfaction. Statistical comparisons were performed using independent samples *t*-tests and χ^2^/Fisher’s tests, with a two-sided *α* = 0.05.

**Results:**

The intervention cohort demonstrated significant improvements in theoretical knowledge (91.1 ± 6.5 vs. 86.5 ± 5.8; mean diff +4.7; *p* < 0.001) and practical skills (92.1 ± 5.4 vs. 82.4 ± 6.1; +9.7; *p* < 0.001), as well as higher scores for learning (+11.4), reasoning/analysis (+12.0), and problem-solving (+11.5) (all *p* < 0.001). Co-curricular and career outcomes also favored the intervention cohort: research participation (50.0% vs. 20.0%), publications (23.3% vs. 3.3%), professional examination pass rates (100% vs. 90%), employment (100% vs. 86.7%), and satisfaction (98.3% vs. 86.7%). However, because the cohorts were not randomized and were drawn from a single center, between-cohort differences may partly reflect selection or cohort effects.

**Conclusion:**

Multicomponent teaching reform was associated with higher knowledge and skills scores and improved co-curricular indicators. Given the non-randomized, single-center, sequential-cohort design, these findings are associative, subject to selection bias, and have limited generalizability. Multisite studies using validated measures are needed to estimate causal impact.

## 1 Introduction

The specialty of medical imaging technology (MIT) is a common major offered by colleges and universities (1, 2). Medical imaging examinations are essential services across all levels of healthcare institutions, creating substantial demand for skilled professionals in this field (3). However, despite the growing demand, MIT education has faced persistent challenges, including outdated curricula, insufficient integration with clinical practice, limited opportunities for simulation-based training, and low levels of student engagement (4–6). These shortcomings limit the ability of graduates to meet the complex demands of modern healthcare, which increasingly values both technical expertise and professional competencies such as clinical reasoning, problem-solving, and teamwork.

In response to these challenges, educational research across health professions has emphasized the need for competency-based education (CBE). CBE prioritizes learning outcomes and measurable competencies over time, encouraging learners to master essential skills through iterative practice and feedback (7). In MIT and related fields, studies have highlighted that blended learning approaches, digital resources, and simulation platforms can significantly improve diagnostic accuracy, technical proficiency, and self-directed learning (8, 9). Moreover, the integration of problem-based learning (PBL), case-based learning, and flipped classrooms has been shown to enhance analytical thinking and clinical decision-making in radiology and allied health education (10–12).

Several reforms in medical and nursing education also provide relevant lessons for MIT. For example, simulation-enhanced curricula in nursing have demonstrated measurable improvements in students’ confidence and readiness for practice (13), whereas structured CBE models in medical technology programs have been associated with greater competency acquisition and smoother transitions to clinical roles (14). However, most existing studies have focused on single interventions, such as flipped classrooms or simulation laboratories, rather than on comprehensive, multicomponent reforms. Consequently, there is limited empirical evidence on the effectiveness of holistic curricular transformations in MIT education.

Our institution sought to address this gap by designing and implementing a multicomponent reform of the MIT curriculum. This reform included the following components: staged competency development (foundation, core competence, and personalized development), enriched curriculum resources [Massive Open Online Course (MOOCs), Small Private Online Course (SPOCs), virtual simulation, and co-developed modules with hospitals], innovative teaching methods (project-based learning, flipped classrooms, and the integration of ideological–political elements), strengthened faculty development, upgraded simulation and clinical practice bases, and a digital skills platform for vocational examination preparation. Unlike traditional lecture-driven models, this reform emphasized experiential learning, interdisciplinary collaboration, and alignment with workforce needs.

To the best of our knowledge, this study is one of the first quasi-experimental evaluations of a comprehensive teaching reform in MIT education in China. By systematically comparing two sequential cohorts, one receiving the traditional curriculum and the other receiving the reformed program, this study provides new empirical evidence on how multilevel teaching innovations can enhance theoretical knowledge, technical skills, higher-order reasoning, and employability among MIT students.

## 2 Materials and methods

### 2.1 Study design and participants

**Figure 1:**
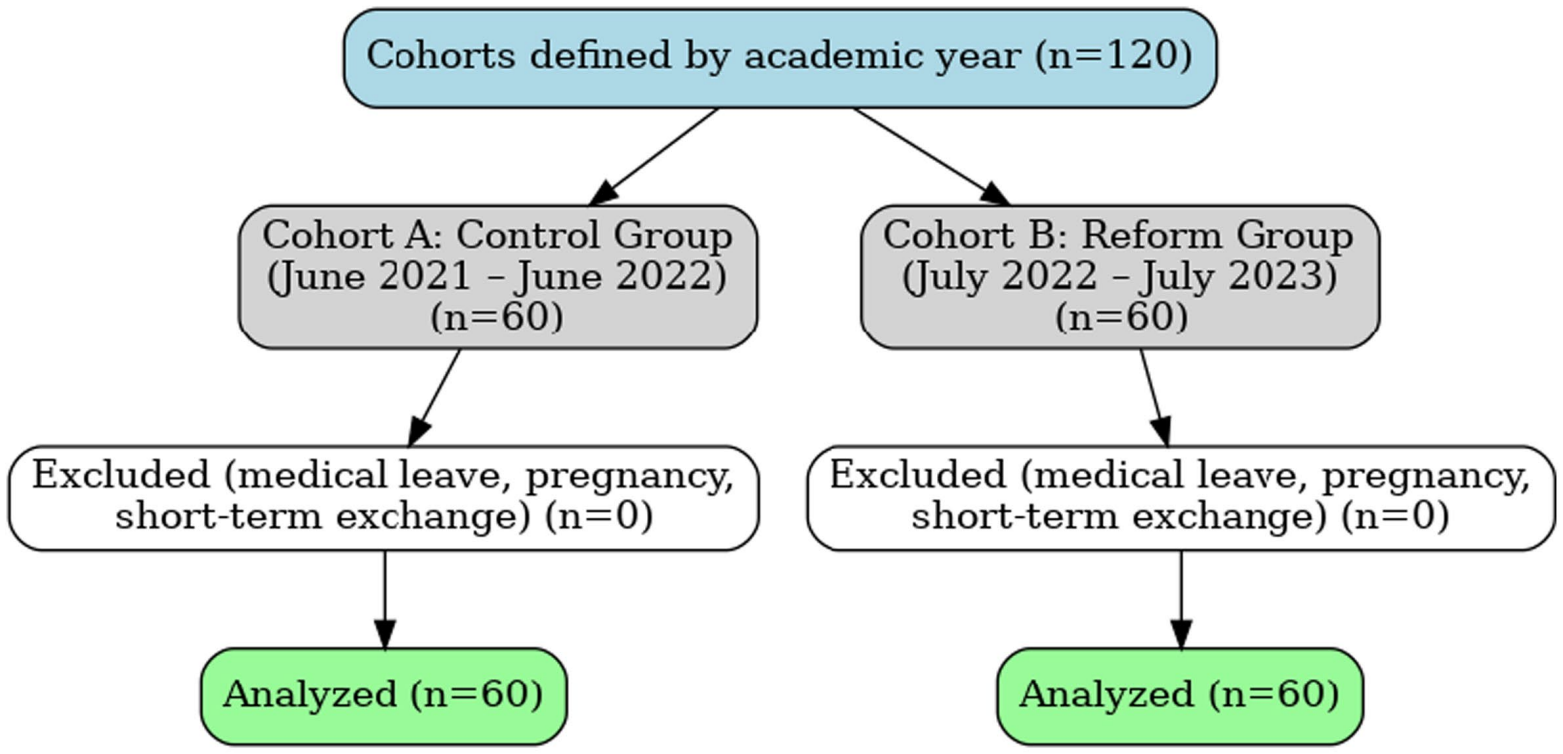
Flow of participants across study cohorts. Cohorts were defined by academic year rather than randomization. Exclusion criteria included medical leave, pregnancy, or short-term exchange; however, no students met these exclusion criteria. All enrolled students were included in the final analysis (*n* = 120).

This study employed a single-center, quasi-experimental, sequential-cohort design in accordance with the Transparent Reporting of Evaluations with Nonrandomized Designs (TREND) guidelines and was conducted at a single institution without random assignment. A control cohort of junior-year MIT students received routine teaching from July 2021 to June 2022 (*n* = 60), followed by an intervention cohort that received the reformed program from July 2022 to July 2023 (*n* = 60). The cohorts corresponded to consecutive academic years; this is noted here once to avoid redundancy. The control group included 22 female and 38 male students (aged 25–28 years; mean 26.17 ± 1.52), and the reform group included 24 female and 36 male students (aged 25–28 years; mean 26.19 ± 1.47). At our institution, junior-year MIT students predominantly enter via a top-up pathway after a 3-year postsecondary diploma or prior clinical employment; therefore, ages 25–28 years are typical for this program. Participant flow, including enrollment, exclusion, and attrition, is summarized in Figure 1.

The study was approved by the Ethics Committee of Baicheng Central Hospital (Approval No. BC20220526991), and written informed consent was obtained from all participants.

The inclusion criteria were as follows: (1) Junior-year students majoring in medical imaging technology at our institution.

The exclusion criteria were as follows: (1) Students on medical leave, (2) pregnant or lactating students, and (3) students participating in short-term exchanges or visiting trainee programs.

### 2.2 Sample size

The sample size was estimated using a two-proportion formula with *α* = 0.05 and *β* = 0.20, using teaching satisfaction as the effect index. Based on a review of the relevant literature and previous studies (15), P1 = 0.95 and P2 = 0.75 were assumed. Following the sample size calculation, the required number of participants was 54 per group. To account for a 10% attrition rate, the target sample size was increased to 60 participants per group (total *N* = 120). The previously repeated “formula” line without content has been removed for clarity.

The formula used for calculating the sample size is shown below:


$$n=[\frac{{\left(Z_{\alpha /2}+Z_{\beta }\right)}^{2}(P_{1}(1-P_{1})+P_{2}(1-P_{2}))}{{\left(P_{1}-P_{2}\right)}^{2}}]$$

### 2.3 Control conditions

Control (“routine teaching”): The control cohort followed the standard MIT curriculum that aligned with the national syllabus and was delivered primarily via lectures (10–12 weeks/term; 4–6 contact hours/week) with two 2-h skills laboratory sessions per month (24 simulation/laboratory h per term). Clinical exposure consisted of a single 4-week hospital placement (40 h/week), which focused mainly on observation, with limited supervised hands-on scanning. Active-learning methods (PBL/flipped) were rarely used (0–5% of sessions) and were not systematically scheduled. Research exposure was opportunistic (no formal pairing; no standing meetings). The assessment comprised a written theory examination (single best answer and short-answer items) and a practical checklist developed by the course faculty without an OSCE blueprint; a single assessor typically scored practical performance, with no formal calibration or inter-rater procedure.

### 2.4 Intervention

**Figure 2:**
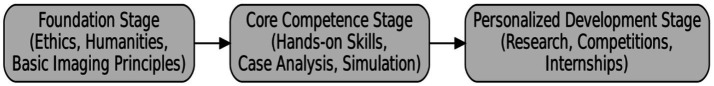
Structured three-stage framework of the teaching reform intervention. The intervention was implemented in three sequential stages. The foundation stage emphasized ethics, humanities, and basic imaging principles. The core competence stage focused on hands-on skills, case analysis, and simulation-based training. The personalized development stage included research activities, competitions, and internships to support individualized professional growth.

The reform cohort received a comprehensive, multicomponent intervention designed to strengthen competency-based training and school–enterprise collaboration. The intervention was structured into three sequential stages: a foundation stage, focusing on ethics, humanities, and imaging principles; a core competence stage, emphasizing hands-on skills, case analysis, and simulation; and a personalized development stage, incorporating research projects, competitions, and clinical internships. A structured summary of the seven components is presented in Table A1, and the staged framework is illustrated in Figure 2. Curriculum resources were enriched through MOOCs, SPOCs, virtual simulations, and co-developed course packages and continuing education modules. Teaching methods and materials were aligned with competency-based and student-centered principles, incorporating flipped classrooms, project-based learning, case discussions, microlectures, and the integration of ideological–political content, as required by policy. An innovation team was established through faculty rotations and external exchanges, mentorship by hospital experts, and regular workshops on curriculum design, teaching methods, and research writing.

Practical training was reinforced through practice bases, including upgraded ultrasound and CT simulation laboratories, structured hospital internships, and the establishment of a virtual–real integrated training center. A technical skills platform was also developed, offering web-based vocational exam preparation, teleimaging support, online tutorials, and mock examinations. Finally, the program promoted social engagement by encouraging participation in professional competitions, community volunteering, and graduate follow-up tracking to monitor employment outcomes.

### 2.5 Outcomes and instruments

To highlight the changes, Table A1 provides a side-by-side comparison of the traditional (“routine”) and reformed curricula. Briefly, the control cohort received lecture-dominant instruction with = 24 simulation hours per term and ≈4 weeks of hospital practice, minimal use of active learning (0–5% PBL/flipped), opportunistic research exposure (no formal pairing/meetings), and an unstructured practical assessment without an OSCE blueprint. In contrast, the reformed program implemented scheduled, OSCE-mapped simulation (72 h/term); PBL/flipped classrooms in 30–50% of sessions; structured research mentorship (faculty:student = 1: K; monthly meetings); competition preparation (three workshops per term); professional exam preparation with two mock exams per term and targeted remediation; expanded clinical exposure (12 weeks); formalized school–hospital modules with supervisor training; faculty development, including peer observation twice per term; and integrated digital resources (MOOCs/SPOCs, virtual simulation, microlectures, and an item bank with analytics). These components represent the key drivers of change believed to account for the observed effects. The primary outcomes were theoretical examination and imaging-skills score (0–100), which were assessed using a structured instrument developed via a two-round Delphi process (five medical-imaging educators; three clinical MIT experts). Content validity was supported by a CVI of 0.90, and internal consistency was demonstrated with a Cronbach’s *α* of 0.91 (subscales 0.81–0.86). The secondary outcomes included learning ability, clinical/analytical reasoning, and problem-solving (each 0–100), measured using a structured questionnaire (*α* = 0.93; subscales 0.84–0.89; CVI = 0.90). Additional indicators included participation in competitions, research projects, and publications; professional qualification exam pass rates; and employment in medical institutions. Overall teaching satisfaction was measured using an institutional 100-point questionnaire (≥70 = “satisfied”; Cronbach’s α = 0.90; CVI = 0.90). As validated MIT-specific instruments were unavailable, institution-developed tools were used, supported by expert validation and reliability testing. We acknowledge that institutionally developed instruments may introduce measurement bias. To improve transparency and enable external appraisal, we have provided the full data-collection instruments as Supplementary material S1: student questionnaire with item stems, response scales, and scoring rules; Supplementary material S2: OSCE station blueprints and checklists with analytic rubrics; and Supplementary material S3: competency mapping, scoring examples, and proctor instructions. To mitigate bias, instruments were refined through a two-round Delphi process, piloted before use, and administered using standardized procedures. For practical examinations, assessors received a calibration guide (Supplementary material S3) and were briefed before each OSCE; two assessors independently scored a random subset of stations, with any discrepancies resolved through discussion according to the rubric. Internal evidence of reliability (Cronbach’s *α*) and content validity (CVI via Delphi) supports score reliability and content coverage but does not constitute external validation. We did not perform multisite psychometric validation (e.g., factor structure, convergent/discriminant validity against established benchmarks, test–retest stability) nor generalizability studies for OSCE scores. The findings should therefore be interpreted with these validation limitations in mind.

Instrument packets are provided in full as Supplementary file. Supplementary material S1: Student questionnaires: Item stems and response scales for learning ability (10 items), clinical reasoning/analysis (10), and problem-solving (10); anchors (1–5), scoring rules (sum → 0–100), and missing-data handling. Supplementary material S2: OSCE materials: Six stations × 8 min each (2-min changeover)—Patient communication and consent, ultrasound probe handling and image optimization, CT safety and protocoling, MRI safety screening, image interpretation (basic chest), and structured reporting template. Each station included a blueprint, a checklist/analytic rubric (item weights, critical fails), standardized instructions, and an examiner timing script. Assessor briefing and calibration notes were also included. Supplementary material S3: Written theory exam blueprint: Domain weights (e.g., physics 20%, safety 15%, cross-sectional anatomy 25%, modality protocols 25%, and professionalism/ethics 15%), item-bank sampling rules (no reuse within the term; ≤20% recall-level items), scoring keys, and standard-setting notes (modified Angoff target ≥70/100) were used. Administration and scoring procedures: Written exams were performed in supervised computer rooms (no internet; 90 min). OSCE examiners were faculty clinicians, with a lead invigilator enforcing timing. Make-up assessments followed the same blueprint. Scoring: Theory exams were auto-scored, OSCE performances were scored on-site and transcribed to a template, and questionnaire subscales were transformed to a 0–100 scale. SPSS v22 was used for analysis (syntax provided in Supplementary material S4).

### 2.6 Statistical analysis

All analyses were performed using SPSS version 22.0. Continuous variables are expressed as means± standard deviations and were compared using independent samples *t*-tests after verifying normality and homogeneity of variance. Categorical variables are expressed as n (%) and were compared using χ^2^ tests, with Fisher’s exact test applied when expected counts were small or when proportions approached 0% or 100%. We report two-sided *p*-values (*α* = 0.05) and effect sizes with 95% confidence intervals (CIs)—mean differences and Cohen’s d values for continuous outcomes, and risk differences and risk ratios for categorical outcomes. Fisher’s exact test was used when cell counts were small or when proportions approached 0% or 100%.

## 3 Results

### 3.1 Comparison of theoretical examination and medical imaging operation skills scores between the two groups

The intervention cohort had higher theoretical and practical scores than the control cohort; these differences should be interpreted as associations given the study design (91.1 ± 6.5 vs. 86.5 ± 5.8; mean diff +4.7, 95% CI +2.3 to +7.0; Cohen’s *d* = 0.77; *p* < 0.001). The intervention cohort also had higher imaging skill scores (92.1 ± 5.4 vs. 82.4 ± 6.1; mean diff +9.7, 95% CI +7.4 to +12.1; *d* = 1.66; *p* < 0.001) (Table 1).

**Table 1:** Comparison of theoretical examination and medical imaging operation skills scores between the two groups.

Group	*n*	Theoretical evaluation score (points)	Operation skills evaluation score (points)	Effect size (theoretical)	95% CI (theoretical)	Effect size (skills)	95% CI (skills)
Control stage	60	86.46 ± 5.78	82.39 ± 6.07	—	—	—	—
Research stage	60	91.13 ± 6.48	92.11 ± 5.44	Mean diff +4.67; *d* = 0.77	+2.35 to +6.99	Mean diff +9.72; *d* = 1.66	+7.37 to +12.07
*t*-value	–	4.166	9.237	–	–	–	–
*p*-value	–	0.000	0.000	–	–	–	–

Two-sided α = 0.05. Fisher’s exact test was applied when expected counts were <5 or proportions approached the ceiling level. Effect sizes are reported as mean differences with 95% CIs and Cohen’s *d*.

Interpretation caveat: These observed differences should be interpreted as associations rather than causal effects because the study used sequential cohorts in a single center without randomization. Unmeasured cohort differences and institutional factors may contribute to the effect sizes reported.

Validity caveat: The outcome instruments were institutionally developed. While the reported Cronbach’s *α* and CVI indicate internal consistency and expert agreement, they do not establish external validity, which may affect the observed effect estimates.

### 3.2 Comparison of learning ability, medical thinking/analytical ability, and problem-solving ability scores between the two groups

The performance cohort outperformed the control cohort in terms of learning ability (92.8 ± 4.6 vs. 81.4 ± 3.5; mean diff +11.4, 95% CI + 9.8 to +13.0; *d* ≈ 2.7; *p* < 0.001), clinical reasoning and analytical ability (92.6 ± 5.1 vs. 80.6 ± 4.6; mean diff +12.0, 95% CI + 10.3 to +13.7; *d* ≈ 2.6; *p* < 0.001), and problem-solving ability (93.8 ± 5.3 vs. 82.3 ± 4.4; mean diff +11.5, 95% CI + 9.8 to +13.2; *d* ≈ 2.5; *p* < 0.001) (Table 2).

**Table 2:** Comparison of learning ability, medical thinking/analytical ability, and problem-solving ability scores between the two groups (with effect sizes and 95% CIs).

Grouping (n)	Learning ability (points)	Medical thinking and analytical ability (points)	Problem-solving ability (points)	Effect size	95% CI
Control stage (*n* = 60)	81.37 ± 3.45	80.57 ± 4.63	82.29 ± 4.37	Ref	–
Research stage (*n* = 60)	92.78 ± 4.58	92.61 ± 5.11	93.81 ± 5.33	Cohen’s *d* ≈ 2.7–3.0	Mean diff ~ + 11 to +12; 95% CI (approx)

Two-sided *α* = 0.05. Fisher’s exact test was used when expected counts were <5 or proportions approached 0%/100%. Effect sizes for continuous outcomes are reported as mean differences with 95% confidence intervals and Cohen’s *d*.

### 3.3 Comparison of award-winning rates, research participation, and publication rates between the two groups in national skills competitions

The intervention cohort had higher award-winning rates (13.3% vs. 1.7%; RD + 11.7%, 95% CI + 1.5% to +22.0%; RR ≈ 8.0; Fisher’s exact *p* = 0.038), research participation (50.0% vs. 20.0%; RD + 30.0%, 95% CI + 15% to +45%; RR = 2.5; *p* < 0.001), and publication rates (23.3% vs. 3.3%; RD + 20.0%, 95% CI + 9% to +31%; RR ≈ 7.0; *p* = 0.001) compared to the control cohort (Table 3).

**Table 3:** Comparison of award rates, research project participation, and publication rates between the two groups (with effect sizes and 95% CIs).

Grouping (n)	Award rate in national skills competition (%)	Participation in research projects (%)	Publication rate (%)	Effect size	95% CI
Control stage (*n* = 60)	1.67	20.00	3.33	Ref	–
Research stage (*n* = 60)	13.33	50.00	23.33	RRs: 8.0, 2.5, 7.0; RDs: +11.7%, +30.0%, +20.0%	95% CI approx ranges (+1.5% to +22%, +15% to +45%, +9% to +31%)
*χ*^2^ value	4.324	11.868	10.385	–	–
P-value	0.038	0.0006	0.001	–	–

Two-sided *α* = 0.05. Fisher’s exact test was used when expected counts were <5 or proportions approached 0%/100%. Effect sizes for categorical outcomes are reported as risk differences (RDs) and risk ratios (RRs) with 95% CIs.

### 3.4 Comparison of technical qualification exam pass rates and employment success rates between the two groups

The exam pass rate was higher in the intervention (reform) group than in the control group (100% [60/60] vs. 90% [54/60]; RD +10.0%, 95% CI +2.4% to +17.6%; RR = 1.11; Fisher’s exact test, *p* = 0.036). The employment success rate was also higher in the intervention group than in the control group (100% [60/60] vs. 86.7% [52/60]; RD +13.3%, 95% CI +5.0% to +22.0%; RR = 1.15; Fisher’s exact test, *p* = 0.010). While these outcomes strongly favored the reform group, the near-ceiling values may partly reflect institutional grading practices, small sample sizes, and the single-center study context and should therefore be interpreted with caution (Table 4). Boundary effect note: Since the intervention cohort reached 100% on these dichotomous outcomes, variability is truncated and estimates lie at the boundary. Small contextual shifts could materially change risk ratios, so these figures should not be overinterpreted as definitive effects of the reform.

**Table 4:** Comparison of health professional qualification exam pass rates and employment success rates between the two groups (with effect sizes and 95% CIs).

Grouping (n)	Qualification exam pass rate (%)	Employment success rate (%)	Effect size	95% CI	Notes
Control stage (*n* = 60)	90.00 (54/60)	86.67 (52/60)	Ref	–	–
Research stage (*n* = 60)	100.00 (60/60)	100.00 (60/60)	RD + 10.0%, RR 1.11 (exam); RD + 13.3%, RR 1.15 (employment)	95% CI approx: exam +1% to +19%, employment +5% to +22%	Fisher’s exact test recommended due to 100% ceiling
*χ*^2^ value	4.386	6.563	–	–	–
*P*-value	0.036	0.010	–	–	–

Two-sided *α* = 0.05. Fisher’s exact test was used when proportions approached 0%/100%. Effect sizes for categorical outcomes are reported as risk differences (RDs) and risk ratios (RRs) with 95% CIs.

### 3.5 Comparison of teaching satisfaction rates between the two groups

Teaching satisfaction was higher in the reform cohort (98.3% vs. 86.7%; RD + 11.7%, 95% CI + 2.0% to +21%; RR = 1.14; Fisher’s exact *p* = 0.038). As with pass rates, these near-perfect satisfaction scores may reflect cultural or institutional factors in addition to reform effects and should therefore be interpreted cautiously (Table 5). Similarly, near-perfect satisfaction constrains variability and reduces the ability to detect true differences, reinforcing the need for careful interpretation of these indicators.

**Table 5:** Comparison of teaching satisfaction rates between the two groups (with effect sizes and 95% CIs).

Grouping (n)	Very satisfied (%)	Relatively satisfied (%)	Dissatisfied (%)	Very dissatisfied (%)	Teaching satisfaction rate (%)	Effect size (95% CI)
Control stage (*n* = 60)	66.67	20.00	8.33	5.00	86.67	Ref
Research stage (*n* = 60)	83.33	15.00	1.67	0.00	98.33	RD + 11.7%, RR 1.14; 95% CI + 2.0% to +21%
*χ*^2^ value	–	–	–	–	4.324	–
*p*-value	–	–	–	–	0.038	–

Two-sided *α* = 0.05. Fisher’s exact test was used when expected counts were <5 or proportions approached 0%/100%. Effect sizes for categorical outcomes are reported as risk differences (RDs) and risk ratios (RRs) with 95% confidence intervals.

## 4 Discussion

As allocation was non-randomized and conducted at a single center, residual selection bias cannot be excluded. With the national health concept increasingly embraced, CT, nuclear magnetic resonance (NMR), ultrasound, and other related imaging techniques have received growing attention. To develop a high-level specialty with MIT characteristics, it is necessary to train skilled professionals in this field (16).

At the same time, international research emphasizes that reforms in health profession education must go beyond policy directives, adopting competency-based, simulation-supported, and innovation-driven approaches that improve workforce readiness (17, 18). Although our intervention aligned with local initiatives such as the “Double High Plan,” this study extends beyond regional policy by providing empirical evidence of improved learning outcomes, consistent with global trends in medical education reform. Similar multistage training frameworks and competency-based curricula in nursing, pharmacy, and radiography have demonstrated improvements in clinical reasoning, procedural competence, and employability (19, 20).

The results indicate that the reform cohort achieved higher scores and superior co-curricular indicators compared to the control cohort. Medical students in the post-reform phase showed higher scores for theoretical examinations, technical proficiency in medical imaging, learning ability, medical reasoning, analytical thinking, and problem-solving skills compared to the control group. Furthermore, the reform cohort demonstrated marked improvements in award rates in national skills competitions, participation in research projects, publication rates, health professional qualification exam pass rates, employment successes in medical institutions, and overall teaching satisfaction. These outcomes are consistent with international reports showing that simulation-based and competency-oriented training is associated with higher technical performance and enhanced higher-order skills (21, 22).

An innovative training model characterized by a course structure that integrates “General Foundation – Core Professional Competencies – Personalized Development,” specifically tailored to the developmental needs of the MIT discipline, has been adopted. The revised talent cultivation framework for high-level MIT-related majors effectively addresses the limitations of traditional teaching approaches, which often place excessive emphasis on general foundational knowledge. This new model facilitates the development of versatile, application-oriented professionals with interdisciplinary competencies, better aligned with the evolving demands of the healthcare industry (21, 22).

Construction of curriculum teaching resources: Emphasis was placed on collaboration with other vocational colleges to broaden and share teaching resources for medical imaging courses, facilitating online instruction. This approach enables medical students to learn flexibly and acquire advanced professional knowledge and operational skills anytime and anywhere, overcoming the constraints of limited curriculum resources and enhancing student satisfaction with teaching (23, 24). Internationally, resource-sharing networks and digital repositories have also been shown to improve accessibility and standardization in radiography and nursing education (25, 26).

Reform of teaching methods and materials: Emphasis was placed on reforming teaching methods and materials, including the selection of superior teaching materials, the adoption of advanced classroom teaching methods, the encouragement of innovative practices, and the promotion of active student participation in topics. These initiatives contribute to the development of comprehensive competencies among medical students, such as learning ability, clinical reasoning, analytical thinking, and problem-solving skills, ultimately leading to increased rates of project participation and paper publication (15, 27). Comparable international reforms, such as flipped classrooms, project-based learning, and integration of ideological or ethical content, have also been associated with enhanced critical thinking and reflective practices among health profession students (28, 29).

Establishment of a teaching innovation team: A dedicated teaching innovation team, composed of experienced educators in high-level vocational education, was established to systematically enhance the quality of training for MIT professionals. This initiative aims to foster pedagogical innovation, strengthen curriculum development, and promote the continuous improvement of teaching practices within the MIT program (30, 31). Similar faculty-development programs internationally have been associated with improved curriculum delivery and the sustainability of reforms (32).

Construction of practical teaching base: A joint practical teaching base was established, including a national standard MIT virtual–real integrated training center. This facilitates hands-on activities for medical students, allowing them to develop professional skills and apply their knowledge in practice, aligning with the teaching goal of “the combination of morality and technology and the combination of work and learning” (33, 34). These practices mirror global advances in simulation-based radiology education, where laboratory-based ultrasound, CT simulators, and hybrid learning environments are recognized for improving safety and competence before clinical placement (35, 36).

Technical skills platform construction: A comprehensive network-based training platform for vocational skills examinations was established, along with a dedicated training base for medical imaging technical personnel. This initiative promotes interdisciplinary collaboration and communication among related majors within the professional group, facilitates the sharing of instructional resources and infrastructure, and strengthens horizontal integration between faculty and students. Collectively, these efforts contribute to improving the overall pass rate in vocational skills examinations and enhancing practical training quality (37). Internationally, digital exam-preparation platforms and e-assessment systems have also been shown to enhance the standardization and fairness of evaluation in medical education (38, 39).

Social services: Long-term collaboration with the teaching hospital, continuous improvement of post-competence, systematic tracking and follow-up of graduates, and measures to ensure high graduate employment rates were implemented (40, 41). Comparable graduate-tracking systems are used internationally to evaluate employability and inform curriculum development, ensuring alignment between training outcomes and healthcare workforce needs (42).

Finally, innovative perspectives must also be acknowledged. Artificial intelligence (AI) is increasingly being applied in radiology education. For example, AI-assisted question generation has been shown to improve the psychometric quality of radiology examinations (43). While our study did not directly integrate AI tools, such approaches represent future directions for MIT education, offering opportunities to enhance assessment, personalize learning, and integrate technological innovation into training.

The near-maximal outcomes observed for professional exam pass rates (100%) and employment (100%) should not be interpreted as definitive effects of the reform. These figures likely reflect a combination of institutional policies (e.g., remediation and targeted exam preparation), local labor market conditions favoring MIT graduates, cultural expectations regarding employment, and sampling constraints in a single cohort. Such ceiling effects reduce measurement sensitivity and can mask between-group differences; they also limit generalizability and may not be reproducible in other settings or larger samples.

In summary, this study contributes to the growing body of evidence showing that competency-based, simulation-supported, and innovation-driven reforms can improve outcomes in medical imaging education. By situating our findings within both local and international contexts, and by highlighting the role of emerging technologies such as AI, we demonstrate the broader relevance and applicability of our reform strategy for health profession education worldwide.

This study addresses the ongoing shift from knowledge-based to competency-based training in health professional education. The multicomponent nature of the reform, which combines curriculum redesign, digital resources and simulation, faculty development, and school–industry collaboration, mirrors the complexity of real educational ecosystems. Our sequential-cohort design with a control year provided a pragmatic comparator when randomization was not feasible. Finally, by evaluating outcomes ranging from basic knowledge and skills to higher-order competencies, research engagement, and career readiness, we offer a holistic perspective on the potential benefits of the reform.

The study has several limitations that warrant consideration. First, the relatively small sample size reduces the statistical power and limits the ability to conduct subgroup analyses. Second, the single-center design restricts the generalizability of the findings beyond our institutional and regional context. To increase the validity and generalizability of future research, it is recommended that subsequent studies adopt a cross-regional, multicenter design with a larger sample population. Such an approach would provide more accurate and comprehensive evidence, thereby improving the applicability of findings to broader clinical and educational settings. Furthermore, the inclusion of structured feedback mechanisms in future investigations could offer valuable insights for refining research methodologies, identifying potential sources of bias, and informing continuous improvement. Several outcomes reached upper bounds (e.g., professional exam pass rates and employment), likely reflecting institutional remediation practices, local hiring demand, and cultural factors rather than the reform alone. Accordingly, these measures have limited discriminatory power, may inflate apparent effects, and should be interpreted cautiously and not assumed to be replicated elsewhere. Dichotomous endpoints that reached 100% in the intervention cohort (exam pass rates and employment) and near-ceiling satisfaction limit variability and statistical power, suppress between-group contrasts, and are highly sensitive to local conditions (e.g., grading/remediation practices and strong regional demand for MIT professionals). Accordingly, these outcomes likely reflect contextual influences as much as intervention effects and should be treated as supportive but non-determinative evidence. Where possible, future studies should analyze continuous or ordinal measures (e.g., scaled exam scores, OSCE subdomain totals, time-to-employment, and contract type) that are less prone to ceiling effects. Another important limitation is the reliance on institution-developed questionnaire measures. Although these tools demonstrated acceptable internal consistency and content validity, the lack of independent external validation introduces the risk of measurement bias and may lead to inflated satisfaction or performance ratings. Moreover, some outcomes reached very high levels (e.g., 100% exam pass rate and 98% satisfaction), which may reflect institutional grading practices, small sample sizes, or cultural factors rather than the sole effect of the intervention. These ceiling effects limit the ability to detect true differences and may overestimate the true impact of the reform. A key limitation is the reliance on institutionally developed instruments rather than externally validated tools. Although we implemented expert review (Delphi), piloting, standardized administration, and assessor calibration (see Supplementary materials S1–S3), residual bias remains possible and effect sizes should be interpreted with caution. Construct validity and external validation: Although internal consistency (*α*) and expert-judged content validity (CVI) were acceptable, these indices, while necessary, are not sufficient for establishing construct validity. We did not perform external validation, including convergent/discriminant validity against established measures, structural validity (factor analysis), test–retest reliability for questionnaires, and inter-rater/generalizability analyses for OSCE performance. Consequently, score interpretations may be imperfect proxies of the intended constructs, and the observed effects could be partly instrument-dependent. Future research should conduct multisite psychometric investigations (e.g., CFA, Rasch/IRT calibration, known-groups validity, and criterion linkage to national examinations) to establish transportable validity evidence. Finally, the quasi-experimental, non-randomized, sequential-cohort design increases the risk of selection bias and overinterpretation. Therefore, the findings should be viewed as associations rather than causal effects. Future multisite studies using validated measures, randomized or controlled designs, and blinded assessments are warranted to confirm and extend these findings.

## 5 Conclusion

The competency-based, simulation-supported teaching reform was associated with higher knowledge and skills scores and improved co-curricular indicators in this single-center, sequential-cohort study. However, near-ceiling outcomes (e.g., 100% exam pass and employment rates) are likely influenced by institutional and contextual factors and should not be interpreted as definitive effects of the reform. Future multisite studies using validated instruments, designs that reduce selection bias, and outcomes less prone to ceiling effects are needed to assess generalizability and estimate causal impact, including formal external validation of outcome instruments.

## Data Availability

The raw data supporting the conclusions of this article will be made available by the authors, without undue reservation.

## Appendix

**Table A1:** Key differences between routine (control) and reformed (intervention) curricula.

Domain	Routine teaching (Control)	Reformed program (Intervention)	Notes/rationale
Contact hours (per term)	Lecture-centric; simulation ≈ 24 h; hospital practice ≈ 4 weeks	Balanced: lectures + active learning; simulation = 72 h; hospital practice = 12 weeks	Report total weekly contact hours for comparability
Simulation and skills labs	*Ad hoc* sessions; limited ultrasound/CT access; no OSCE blueprint	Scheduled blocks; upgraded ultrasound/CT suites; OSCE-style stations mapped to competencies; checklists used	Links to primary outcome (skills 0–100)
Active learning methods	Rare PBL/flipped classroom use (≈ 0–5% of sessions)	Routine PBL/case discussion; flipped classrooms in ≈ 30–50% of sessions; project-based modules each term	Specify proportions by course if available
Digital/online resources	Basic PPT + LMS uploads	MOOCs/SPOCs integrated; virtual simulation platform; micro-lectures; question bank and analytics	Name platforms if journal permits
Assessment	Midterm/final written exams; practical unstructured	Written exams + OSCE-style practical; frequent low-stakes quizzes; rubric-based case analyses	Aligns with reported outcomes
Research mentorship	Opportunistic; no formal pairing	Formal mentor assignment (faculty: student = 1: K); scheduled monthly research meetings; deliverables (proposal → data → poster/manuscript)	Tie to research/publication rates
Competition preparation	Self-initiated	Institutional preparation workshops (3 per term); practice judging; travel support	Matches competition award outcomes
Professional exam preparation	Self-study	Digital skills platform; mock exams (2 per term); targeted remediation	Supports 100% pass rates
Industry/clinical collaboration	Informal contacts	Signed school–hospital modules; defined competencies by rotation; supervisor training	Strengthens internship quality
Faculty development	None/minimal	Workshops on CBE, flipped classroom/PBL, OSCE; peer observation rounds (2 per term)	Mechanism for fidelity
Ideological–political elements	Implicit	Explicit integration via case prompts and reflection	Per policy requirement

Percentages and counts reflect course logs and schedules for the relevant academic years, where available.
