# Supplementary material for: Competency-based, multicomponent teaching reform in medical imaging technology: a quasi-experimental study
Source: Front Med (Lausanne). 2026 Jan 12;12:1708856. doi: 10.3389/fmed.2025.1708856 (PMC12832869; doi:10.3389/fmed.2025.1708856)
Supplement: Supplementary file 1 [file Supplementary_file_1.docx]

**Supplementary S1.**

**Student Questionnaire: Items, Response Options, and Scoring Rules**

Overview: This questionnaire measures learning ability, clinical/analytical reasoning, problem-solving, and satisfaction. Use 5-point Likert scales unless otherwise specified.

**Instructions for respondents:**

Please read each statement and select the option that best reflects your experience in this course. There are no right or wrong answers.

Response scale (ability/reasoning/problem-solving): 1 = Strongly disagree, 2 = Disagree, 3 = Neutral, 4 = Agree, 5 = Strongly agree.

Response scale (satisfaction): 1 = Very dissatisfied, 2 = Dissatisfied, 3 = Neutral, 4 = Satisfied, 5 = Very satisfied.

| Learning Ability (LA) Items | Response (1–5) | Notes |
| --- | --- | --- |
| [Learning-1] I plan my study activities effectively. | 1 2 3 4 5 |  |
| [Learning-2] I regularly review course content before labs. | 1 2 3 4 5 |  |
| [Learning-3] I can identify my own learning gaps and address them. | 1 2 3 4 5 |  |

| Clinical/Analytical Reasoning (CR/AR) Items | Response (1–5) | Notes |
| --- | --- | --- |
| [Clinical/Analytical-1] I can select appropriate imaging modalities for common clinical scenarios. | 1 2 3 4 5 |  |
| [Clinical/Analytical-2] I can justify parameter choices for routine protocols. | 1 2 3 4 5 |  |
| [Clinical/Analytical-3] I can interpret common imaging findings and suggest next steps. | 1 2 3 4 5 |  |

| Problem-Solving (PS) Items | Response (1–5) | Notes |
| --- | --- | --- |
| [Problem-Solving-1] When faced with unfamiliar equipment behavior, I can troubleshoot systematically. | 1 2 3 4 5 |  |
| [Problem-Solving-2] I adapt to unexpected patient factors during imaging. | 1 2 3 4 5 |  |
| [Problem-Solving-3] I can synthesize information from multiple sources to make decisions. | 1 2 3 4 5 |  |

| Satisfaction (SAT) Items | Response (1–5) | Notes |
| --- | --- | --- |
| [Satisfaction-1] Overall, I am satisfied with the course organization. | 1 2 3 4 5 |  |
| [Satisfaction-2] The simulation components met my learning needs. | 1 2 3 4 5 |  |
| [Satisfaction-3] The feedback I received was timely and useful. | 1 2 3 4 5 |  |

**Scoring and composites:**

Compute the mean (or sum) for each subscale (LA, CR/AR, PS, SAT). Transform to 0–100 if required. Reverse-coded items: none. Missing data rule: compute if ≥80% answered.

**Reliability/validity notes:**

Internal consistency observed in the study cohort: α=0.93 (subscales 0.84–0.89). Content validity (CVI=0.90) from a two-round Delphi with educators and clinical experts.

**Supplementary S2.**

**OSCE Station Blueprints, Checklists, and Analytic Rubrics**

Version date: 2025-11-04

Overview: Four OSCE stations mapped to core MIT competencies. Each station includes objectives, materials, timing, checklist items, and an analytic rubric with a global rating.

**Station 1: Ultrasound Scanning Technique**

Objectives:

• Prepare the patient and equipment safely.

• Select and optimize transducer, depth, gain, and focus.

• Acquire standard images for the target organ.

Materials: Ultrasound simulator or phantom; gel; wipes; instruction sheet.

Timing: 10 minutes (2-min setup, 7-min task, 1-min cleanup)

| Checklist Item | Score |
| --- | --- |
| Performs hand hygiene and patient ID check (0/1) | 0 / 1 |
| Applies gel and selects appropriate transducer (0/1) | 0 / 1 |
| Optimizes depth/gain/focus appropriately (0/1) | 0 / 1 |
| Acquires required planes (longitudinal/transverse) (0/1) | 0 / 1 |
| Saves/labels images correctly (0/1) | 0 / 1 |

| Analytic Rubric Criterion | Score (0–2) |
| --- | --- |
| Image optimization (0–2) | 0 1 2 |
| Anatomic coverage (0–2) | 0 1 2 |
| Technical efficiency (0–2) | 0 1 2 |
| Patient safety & communication (0–2) | 0 1 2 |
| Professionalism (0–2) | 0 1 2 |

Global rating (1–5): 1=Unsatisfactory, 3=Borderline, 5=Excellent. Pass standard: checklist ≥4/5 AND global ≥3.

Assessor note: Provide narrative feedback for any criterion scored 0.

**Station 2: CT Safety and Protocoling**

Objectives:

• Confirm indication and assess contraindications.

• Select protocol parameters (kVp, mAs, slice thickness) aligned with ALARA.

• Explain contrast use and consent requirements.

Materials: CT console simulator; protocol sheets; contrast policy; mock consent form.

Timing: 10 minutes

| Checklist Item | Score |
| --- | --- |
| Reviews indication and checks contraindications (0/1) | 0 / 1 |
| Chooses parameters appropriate to BMI/indication (0/1) | 0 / 1 |
| Applies radiation safety principles (ALARA) (0/1) | 0 / 1 |
| Explains contrast risks and obtains mock consent (0/1) | 0 / 1 |
| Documents protocol clearly (0/1) | 0 / 1 |

| Analytic Rubric Criterion | Score (0–2) |
| --- | --- |
| Parameter selection rationale (0–2) | 0 1 2 |
| Safety and policy adherence (0–2) | 0 1 2 |
| Clarity of patient explanation (0–2) | 0 1 2 |
| Documentation quality (0–2) | 0 1 2 |
| Efficiency/time management (0–2) | 0 1 2 |

Global rating (1–5): 1=Unsatisfactory, 3=Borderline, 5=Excellent. Pass standard: checklist ≥4/5 AND global ≥3.

Assessor note: Provide narrative feedback for any criterion scored 0.

**Station 3: Image Interpretation & Reporting**

Objectives:

• Identify key findings on provided images.

• Prioritize differential diagnoses.

• Compose a concise, structured report.

Materials: PACS viewer or printed images; structured reporting template.

Timing: 10 minutes

| Checklist Item | Score |
| --- | --- |
| Systematic search pattern demonstrated (0/1) | 0 / 1 |
| Correctly identifies primary finding(s) (0/1) | 0 / 1 |
| Provides reasonable differential (0/1) | 0 / 1 |
| Recommends appropriate next step (0/1) | 0 / 1 |
| Completes structured report elements (0/1) | 0 / 1 |

| Analytic Rubric Criterion | Score (0–2) |
| --- | --- |
| Diagnostic accuracy (0–2) | 0 1 2 |
| Reasoning/justification (0–2) | 0 1 2 |
| Report structure & clarity (0–2) | 0 1 2 |
| Recommendations (0–2) | 0 1 2 |
| Professionalism (0–2) | 0 1 2 |

Global rating (1–5): 1=Unsatisfactory, 3=Borderline, 5=Excellent. Pass standard: checklist ≥4/5 AND global ≥3.

Assessor note: Provide narrative feedback for any criterion scored 0.

**Station 4: Patient Positioning & Communication**

Objectives:

• Demonstrate safe positioning for a given exam.

• Communicate effectively with the patient.

• Adjust technique for patient-specific limitations.

Materials: Positioning aids; mock patient or mannequin; scenario card.

Timing: 10 minutes

| Checklist Item | Score |
| --- | --- |
| Verifies identity and explains procedure (0/1) | 0 / 1 |
| Positions patient safely with appropriate supports (0/1) | 0 / 1 |
| Checks comfort/pain and adjusts as needed (0/1) | 0 / 1 |
| Maintains privacy and dignity (0/1) | 0 / 1 |
| Documents positioning and precautions (0/1) | 0 / 1 |

| Analytic Rubric Criterion | Score (0–2) |
| --- | --- |
| Biomechanics/safety (0–2) | 0 1 2 |
| Adaptability (0–2) | 0 1 2 |
| Communication (0–2) | 0 1 2 |
| Patient-centeredness (0–2) | 0 1 2 |
| Overall efficiency (0–2) | 0 1 2 |

Global rating (1–5): 1=Unsatisfactory, 3=Borderline, 5=Excellent. Pass standard: checklist ≥4/5 AND global ≥3.

Assessor note: Provide narrative feedback for any criterion scored 0.

**Supplementary S3.**

**Competency Mapping, Scoring Examples, Assessor Calibration, and Administration SOPs**

Version date: 2025-11-04

This document links learning outcomes to instruments, provides scoring examples, outlines assessor calibration, and details OSCE administration procedures.

Competency mapping:

| Learning Outcome | Questionnaire Subscale/Items | OSCE Station(s) | Evidence/Notes |
| --- | --- | --- | --- |
| Foundational knowledge | LA items, CR/AR-1 | Station 2, Station 3 | Protocol rationale; interpretation accuracy |
| Hands-on imaging skills | PS-2, PS-3 | Station 1, Station 4 | Optimization; positioning; safety |
| Clinical reasoning | CR/AR items | Station 2, Station 3 | Differential diagnosis; next-step planning |
| Professionalism/communication | SAT-3 | Station 4 | Patient explanation; privacy/dignity |

**Scoring examples:**

Example 1 (Questionnaire): LA items = 4, 5, 3 → mean = 4.0; scaled to 0–100 = 75.

Example 2 (OSCE Station 1): Checklist 5/5; rubric total 8/10; global = 4/5 → Pass (meets both thresholds).

**Assessor calibration protocol (pre-OSCE, ~20 minutes):**

• Review station objectives, checklists, and rubrics; agree on behavioral anchors for 0/1 and 0–2 scales.

• Jointly score exemplar performances; discuss discrepancies; record anchor decisions.

• Define rules for borderline credit and safety-critical failures.

• Provide a one-page crib sheet at each station summarizing anchors.

**Administration SOPs:**

• Timing: 10 minutes per station; 1-minute transition; 4 stations total.

• Roles: assessor per station; timekeeper; proctor; equipment runner.

• Materials: station instructions, checklists, rubrics, consent forms (mock), ultrasound gel/wipes, positioning aids.

• Data: candidate ID and station code on every sheet; double entry of totals; secure storage.

• Disruptions: pause only for safety; document incident details; inform lead examiner.

• Post-exam: collect materials; reconcile scores; store documents in a secure folder.

**Discrepancy resolution:**

Two assessors independently co-score a 10% random sample of stations; discrepancies ≥2 rubric points trigger review and consensus.

**Data management and privacy:**

De-identify all student artifacts; store consent forms separately; restrict database access to authorized staff.
